# Arginine Vasotocin and Cortisol Co-regulate Vasotocinergic, Isotocinergic, Stress, and Thyroid Pathways in the Gilthead Sea Bream (*Sparus aurata*)

**DOI:** 10.3389/fphys.2019.00261

**Published:** 2019-03-21

**Authors:** Juan Antonio Martos-Sitcha, Laura Cádiz, Magdalena Gozdowska, Ewa Kulczykowska, Gonzalo Martínez-Rodríguez, Juan Miguel Mancera

**Affiliations:** ^1^Department of Biology, Faculty of Marine and Environmental Sciences, Instituto Universitario de Investigación Marina, Campus de Excelencia Internacional del Mar, University of Cádiz, Cádiz, Spain; ^2^Department of Marine Biology and Aquacuture, Instituto de Ciencias Marinas de Andalucía, Consejo Superior de Investigaciones Científicas, Cádiz, Spain; ^3^Department of Genetics and Marine Biotechnology, Institute of Oceanology, Polish Academy of Sciences, Sopot, Poland

**Keywords:** arginine vasotocin, cortisol, Crh, isotocin, receptors, *Sparus aurata*, stress, thyroid system

## Abstract

In teleosts, a complex interaction between several endocrine axes modulates physiological functions related to metabolism, stress, and osmoregulation. Although many studies in fish underline the interconnection between the hypothalamic–pituitary–interrenal (HPI) and hypothalamic–pituitary–thyroid (HPT) endocrine axes, their relationship with the vasotocinergic and isotocinergic systems remains unknown. The aim of the present study is therefore to shed light on the potential cross-regulations between HPT, HPI, and the vasotocinergic and isotocinergic axes in gilthead sea bream (*Sparus aurata*) at hypothalamic, hypophyseal, and plasma levels. Sea breams were administered with intraperitoneal slow-release implants containing different doses of vasotocin (the active peptide in vasotocinergic system) or cortisol (the last component of HPI axis). Plasma osmolality was higher in active neuropeptides vasotocin (Avt)-treated fish, indicating an osmoregulatory function of this hormone. Low concentrations of Avt increased hypothalamic *arginine vasotocin precursor* (*avt*) mRNA levels and increased Avt storage in the pituitary. Avt treatment down-regulated hypothalamic *arginine vasotocin receptor v1a-type* (*avtrv1a*), suggesting a negative paracrine co-regulation of the HPI axis due to the close location of *avtrv1a* and adrenocorticotropin hormone (Acth) cells in the anterior pituitary. Furthermore, the up-regulation observed in *arginine vasotocin receptor v2-type* (*avtrv2*) suggests their involvement in metabolic and cortisol-related pathways in the hypothalamus. The decrease in isotocin (It) pituitary storage and the up-regulation of *it* receptor, observed in the Avt-treated group, reinforce the idea of an interconnection between the vasotocinergic and isotocinergic systems. Cortisol and Avt administration each inhibited the HPI axis, down-regulating *crh* gene expression in the absence of variations in *corticotropin releasing hormone binding protein* (*crhbp*). Finally, both hormonal treatments activated the HPT axis via up-regulation of *trh* and down-regulation of *thrb*. Our results provide evidence for strong interactions among the Avt/It, HPI, and HPT axes of marine teleosts, particularly at the hypothalamic level.

## Introduction

The endocrine system is considered a complex net of interconnected pathways, and establishing the relevance of these pathways is critical for a correct understanding of their putative relationships. In response to stressors, the hypothalamus–pituitary–interrenal (HPI) axis is activated in a coordinated manner leading to the release of cortisol (Wendelaar Bonga, 1997). Cortisol is the main corticosteroid in teleosts and its function is related to growth, intermediary metabolism, osmotic and ionic regulation, stress, and immunity (Henderson and Garland, 1980; McCormick, 1995; Mommsen et al., 1999; Mancera and McCormick, 2007; Sherwani and Parwez, 2008). The release of cortisol is controlled by the hypothalamic corticotrophin-releasing hormone (Crh) and the Crh binding protein (Crhbp). Specifically, Crh stimulates the release of the adrenocorticotropin hormone (Acth), which is cleaved from the precursor protein proopiomelanocortin (Pomc), produced in adenohypophyseal corticotroph cells. Subsequently, Acth activates cortisol biosynthesis and releases in the interrenal cells dispersed throughout the head kidney (Wendelaar Bonga, 1997; Flik et al., 2006; Aluru and Vijayan, 2008; Bernier et al., 2009). Moreover, Crhbp is considered an antagonist of the Crh function, due to the higher affinity of Crh for Crhbp than for Crh receptors (Huising et al., 2004).

The thyroidal [hypothalamic–pituitary–thyroid (HPT)] axis is well known in mammals (Sower et al., 2009), but in fish, many aspects of its control mechanisms remain unclear (De Groef et al., 2006). Current studies indicate that the thyrotrophin-releasing hormone (Trh) controls the release of the thyroid-stimulating hormone (Tsh) in teleosts, which in turn stimulates the thyroid gland to synthesize and secrete thyroid hormones (THs) [thyroxine (T4) and triiodothyronine (T3)] (Chatterjee et al., 2001; Han et al., 2004). In teleost, THs have a diverse array of physiological functions such as osmoregulation, nutrition, metabolism, and reproduction, among others (see Gorbman et al., 1983; Norris, 1997).

The interrelationship between the HPT and HPI axes in teleosts has been the subject of many studies (Dange, 1986; Redding et al., 1986; Shrimpton and McCormick, 1998). Thus, in brook charr (*Salvelinus fontinalis*), *in vitro* studies with liver homogenates demonstrated that cortisol exposure increased the hepatic conversion of T4 to T3 (Vijayan and Leatherland, 1988). In Japanese flounder (*Paralichthys olivaceus*), cortisol enhanced the effects of both T4 and T3 on the resorption of the dorsal fin ray (de Jesus et al., 1990). More recently, Geven et al. (2009) demonstrated that cortisol and Acth can stimulate the release of T4 from renal tissues, and T4 can inhibit the HPI axis via Crhbp in the preoptic area in the common carp (*Cyprinus carpio*). Furthermore, in freshwater tilapia (*Oreochromis mossambicus*), exogenous T3 activated the interrenal axis to produce cortisol (Peter and Peter, 2009).

In teleosts, physiological processes such as osmoregulation, metabolism, or stress, in which HPI and HPT axes play important roles, are also mediated by the vasotocinergic and isotocinergic systems, which are homologous to the mammalian vasopressinergic and oxytocinergic pathways (Warne et al., 2002; Balment et al., 2006; Kulczykowska, 2007). However, to the best of our knowledge, the physiological interactions between the HPI and HPT axes and the vasotocinergic and isotocinergic pathways at the hypothalamic level remain unknown in fish. The vasotocinergic and isotocinergic systems are controlled by many external and internal factors, which start an endocrine process for the production of the active neuropeptides vasotocin (Avt) and isotocin (It) in different hypothalamic nuclei, with the preoptic and lateral tuberal nuclei considered the most important. Avt and It are then transported to the neurohypophysis for storage and releases into the bloodstream in response to specific stimulation (Goossens et al., 1977; Peter, 1977; van den Dungen et al., 1982). Specifically, Avt has been demonstrated to orchestrate osmoregulatory processes related to hyperosmotic environments, control of blood pressure and cardiovascular activity, reproductive behavior, brain neurotransmission, and pituitary endocrine activity (Warne et al., 2002; Balment et al., 2006; Kulczykowska, 2007). Even if the role for It is less clear, previous studies related this hormone to social status and behavior in fish (Almeida et al., 2012; Kleszczyńska et al., 2012). Moreover, recent studies in marine teleosts demonstrated that both endocrine pathways are present and affected in important tissues involved in the regulation of several acute and chronic stress events, as osmotic challenges, starvation, high stocking densities, or air exposure (Martos-Sitcha et al., 2013b, 2014a; Skrzynska et al., 2017, 2018a,b).

The purpose of this work is to unravel the interaction between the HPT and HPI axes and the vasotocinergic and isotocinergic systems in the gilthead sea bream (*Sparus aurata*). This species was selected as a biological model because of the available data on the endocrine mechanisms involved in stress, osmoregulation, and metabolism (Arends et al., 1999; Laiz-Carrión et al., 2002, 2005; Mancera et al., 2002; Sangiao-Alvarellos et al., 2003; Martos-Sitcha et al., 2014b; Ruiz-Jarabo et al., 2018), and due to its importance in Mediterranean aquaculture. The first specific aim was to evaluate, in a short-time course response, hypothalamic components of the vasotocinergic, isotocinergic, HPI, and HPT axes following an intraperitoneal administration of slow-release implants containing different doses of Avt or cortisol. The subsequent objective was to determine the dynamic of changes of hypophyseal Avt/It content as well as its consequences regarding their release, concomitantly with cortisol hormone, into the blood stream. Finally, new insights into the regulation and interconnection between different physiological and endocrine pathways are also provided.

## Materials and Methods

### Animals and Experimental Protocol

Gilthead sea bream (*S. aurata* L., 193.2 ± 3.5 g body mass) juveniles were obtained from fish-farming sources (CUPIMAR, San Fernando, Cádiz, Spain) and provided by Servicios Centrales de Investigación en Cultivos Marinos (SCI-CM, CASEM, University of Cádiz, Cádiz, Spain; Operational Code REGA ES11028000312). Animals were then randomly distributed to 400-L tanks (density ∼3.5 kg ⋅ m^-3^), in an open system circuit, and acclimated for 14 days to seawater (SW, 38‰ salinity), during the natural photoperiod (February–March) for the latitude (36° 31’ 44” N) and constant temperature (18–19°C) at the indoor wet laboratories in the Faculty of Marine and Environmental Sciences (Puerto Real, Cádiz, Spain). Fish were fed a daily ration of 1% of their body mass with commercial pellets (Dibaq-Dibroteg S.A., Segovia, Spain). All procedures were approved by the Ethics and Animal Welfare Committee of the University of Cádiz and carried out according to the Guidelines of the European Union (2010/63/UE) and the Spanish legislation (RD53/2013) for the use of laboratory animals.

#### Experiment I: Avt Administration

On day 0, experimental fish (*n* = 63) allocated in six 400-L tanks were lightly anesthetized with 2-phenoxyethanol (0.5 mL ⋅ L^-1^ SW), weighed, and intraperitoneally injected (two tanks per experimental condition) with a mixture of coconut and sunflower oils (in a proportion 5:1, 5 μL ⋅ g^-1^ body mass) alone (control, *n* = 21) or containing different doses (0.5 or 1.0 μg ⋅ g^-1^ body mass, *n* = 21 per dose) of Avt (Arg^8^-vasotocin; Sigma, Cat. #V0130). These implants can function in a fish body for a continuous release over at least 2–6 days (Sangiao-Alvarellos et al., 2006). 2-Phenoxyethanol used here and elsewhere was selected due to its short time to induce deep anesthesia (less than 1 min) without significant effects on the physiological parameters assessed (Toni et al., 2015; Priborsky and Velisek, 2018). Later, fish were recovered in their respective experimental units and maintained at the same environmental conditions as described above until sampling procedures. Both doses of Avt used have previously been shown to be effective in the activation of endocrine and osmoregulatory pathways (Sangiao-Alvarellos et al., 2006). No mortality was observed in any of the experimental groups.

#### Experiment II: Cortisol Administration

Experimental design was performed as previously described by Cádiz et al. (2015). In short, on day 0, experimental fish (*n* = 42) from six 400-L tanks were lightly anesthetized with 2-phenoxyethanol (0.5 mL ⋅ L^-1^ SW), weighed, and intraperitoneally injected (in triplicate tanks) with a mixture of coconut and sunflower oils (in a proportion 5:1, 5 μL ⋅ g^-1^ body mass) alone (control, *n* = 21) or containing cortisol (50 μg ⋅ g^-1^ body mass, *n* = 21) (hydrocortisone 21-hemisuccinate sodium salt, Sigma–Aldrich, Cat. No. H2270, Madrid, Spain), and recovered as described above. No mortality was observed in any of the experimental groups.

### Sample Collection

Seven fish from each experimental condition (at Experiments I and II) were deeply anesthetized with 2-phenoxyethanol (1 mL ⋅ L^-1^ SW), weighed, and sampled (blood, pituitary, and hypothalamus) at 12 h, 1 and 3 days after hormone administration. In addition, seven to eight fish from non-treated groups were sampled on day 0 as an extra control. Blood samples were collected from the caudal blood vessels into 2 mL ammonia-heparinized (Sigma) syringes, and centrifuged (3 min at 10,000 *g*). Plasma samples were divided into two Eppendorf tubes, for measurement of Avt, It, cortisol, and osmolality. Pituitaries were snap-frozen in liquid nitrogen and stored at -80°C. The two complete hypothalamic lobules (including all neuronal regions contained in this brain division) were put in a 1/10-relation w/v of RNA*later*^TM^ stabilization solution (Ambion^®^) for 24 h at 4°C and then stored at -20°C until total RNA isolation.

### Plasma Parameters

Plasma osmolality was measured from 10 μL of each individual sample with a vapor pressure osmometer (Fiske One-Ten Osmometer, Fiske-VT, United States) and expressed as mOsm ⋅ kg^-1^.

Plasma cortisol levels were measured by enzyme-linked immunosorbent assay (EIA) adapted to microtiter plates as previously described by Martos-Sitcha et al. (2014b) for this species. In short, steroids were extracted from 5 μL of plasma, and cortisol EIA standard (Cat. #10005273), goat anti-mouse IgG monoclonal antibody (Cat. #400002), cortisol monoclonal antibody (Cat. #400372), and cortisol-AChE tracer (Cat. #400370) were obtained from the Cayman Chemical Company (Michigan, United States). Standards and extracted plasma samples were run in duplicate. A standard curve was run from 2.5 ng/mL to 9.8 pg/mL (*R*^2^ = 0.992). The lower limit of detection (98.8% of binding, ED98.8) was 10.1 pg/mL. The percentage of recovery was 95.0%. The inter- and intra-assay coefficients of variation (calculated from the sample duplicates) were 0.8 ± 0.11 and 3.5 ± 0.55%, respectively. The cross-reactivity of the specific antibody toward intermediate steroid synthesis products was determined by the supplier [Cayman Chemical Company, Michigan, United States; cortexolone (1.6%), 11-deoxycorticosterone (0.23%), 17-hydroxyprogesterone (0.23%), cortisol glucuronide (0.15%), corticosterone (0.14%), cortisone (0.13%), androstenedione (<0.01%), 17-hydroxypregnenolone (<0.01%), testosterone (<0.01%)].

### Quantification of mRNA Expression Levels

In all protocols, the manufacturer’s instructions were followed except where stated. Total RNA was extracted using an Ultra-Turrax^®^ T8 (IKA^®^-Werke) from both hypothalamic lobes using the NucleosSpin^®^ RNA kit (Macherey-Nagel) in a volume of 600 μL of buffer RA1 contained in the kit. Genomic DNA was removed by the on-column DNase digestion at 37°C for 30 min, using the rDNase (RNase-free) included in the kit. RNA quality was checked in an Agilent 2100 Bioanalyzer and with the Agilent RNA 6000 Nano Assay kit (Agilent Technologies), whereas RNA quantity was fluorimetrically measured with the Qubit^®^ 2.0 Fluorometer (Invitrogen^TM^, Life Technologies). All samples processed presented an RNA Integrity Number (RIN) higher than 8.50, which was indicative of the intact RNA to be used for real-time quantitative PCR (qPCR). Primers for *arginine vasotocin precursor* (*avt*, acc. no. FR851924), *isotocin* (*it*, acc. no. FR851925), *arginine vasotocin receptor v1a-type* (*avtrv1a*, acc. no. KC195974), *arginine vasotocin receptor v2-type* (*avtrv2*, acc. no. KC960488), *isotocin receptor* (*itr*, acc. no. KC195973), *crh* (acc. no. KC195964), *crhbp* (acc. no. KC195965), *trh* (ac. no.: KC196277), *thyroid hormone receptor beta* (*thrb*, acc. no. AY246695), and *beta actin* (*actb*, acc. no. X89920) from *S. aurata* (at the final concentration provided in Supplementary Table S1) were used as previously described by Martos-Sitcha et al. (2013b, 2014a,b) and Ruiz-Jarabo et al. (2018). Several calibration plots with different template concentrations in serial dilutions (from 10 ng to 100 fg of input total RNA) had amplification efficiencies and *r*^2^ of 0.97–1.01 and 0.99–1.00, respectively, for all primer pairs used. Each reaction mixture (10 μL) contained 0.5 μL of each specific forward and reverse primers concentration, and 5 μL of PerfeCTa SYBR^®^ Green FastMix^TM^ (Quanta Biosciences). Reactions were conducted in semi-skirted twin.tec real-time PCR plates 96 (Eppendorf) covered with adhesive Masterclear real-time PCR Film (Eppendorf), and run in an Eppendorf Mastercycler ep realplex^2^ S. The PCR profile was as follows: 95°C, 10 min; [95°C, 30 s; 60°C, 45 s] × 40 cycles; *melting curve* [60–95°C, 20 min], 95°C, 15 s. The melting curve was used to ensure that a single product was amplified and to check for the absence of primer-dimer artifacts. Results were normalized to *S. aurata* β-actin (*actb*, X89920) owing to its low variability (less than 0.3 C_T_, ranged between 13.81 and 14.07 C_T_) among fish of both experimental conditions and sub-trials. Relative gene quantification was performed using the ΔΔC_T_ method (Livak and Schmittgen, 2001). This manuscript follows the ZFIN Zebrafish Nomenclature Guidelines for gene and protein names and symbols^1^.

### Avt and It Content in Plasma and Pituitary

The Avt and It content in the plasma and pituitary gland were determined using high-performance liquid chromatography (HPLC) with fluorescence detection, preceded by solid-phase extraction (SPE) based on Gozdowska et al. (2006) and Martos-Sitcha et al. (2013b). Thus, plasma samples (1 mL each) were acidified with 1 M HCl (100 μL) and centrifuged at 6000 *g* for 20 min at 4°C, whereas frozen pituitaries were weighed (441 ± 11 μg) and sonicated in 0.5 mL Milli-Q water (Microson^TM^XL, Misonix, United States). After that, the SPE protocol was carried out for the Avt and It extraction. The resulting eluate was evaporated to dryness using a Turbo Vap LV Evaporator (Caliper Life Scence, United States) and samples were frozen and stored at -80°C until HPLC analysis. The samples were then re-suspended in 40 μL 0.1% trifluoroacetic acid (TFA) and then divided into two aliquots for duplicate analysis. Pre-column derivatization of Avt and It in 20 μL samples was performed using 3 μL 4-fluoro-7-nitro-2,1,3-benzoxadiazole (NBD-F) solution (30 mg NBD-F in 1 mL of acetonitrile) in a mixture of 20 μL phosphate buffer (0.2 M, pH 9.0) and 20 μL acetonitrile. The solution was heated to 60°C for 3 min in a dry heating block and cooled on ice. Next, 4 μL 1 M HCl was added. Derivatized samples were measured using an Agilent 1200 Series Quaternary HPLC System (Agilent Technologies, United States). Chromatographic separation was achieved using Agilent ZORBAX Eclipse XDB-C18 columns (150 mm × 4.6 mm I.D., 5 μm particle size). A gradient elution system was used to separate the derivatized peptides. The mobile phase consisted of solvent A (0.1 % TFA in H_2_O) and solvent B (0.1 % TFA in acetonitrile: H_2_O (3:1). A linear gradient was 45–70% eluent B in 20 min. Flow rate was set at 1 mL/min and the column temperature at 20°C. The injection volume was 67 μL. Fluorescence detection was carried out at 530 nm with excitation at 470 nm.

### Statistics

After the normality and homogeneity of variances where checked, statistical differences were analyzed by two-way ANOVA with treatment (control, hormone administration) and time (day 0, 12 h, days 1 and 3) as main factors. These analyses were followed by Tukey’s test *post hoc* comparisons using GraphPad Prism^®^ (v.5.0b) software. Statistical significance was accepted at *P* ≤ 0.05. Statistical parameters (*P*-value and *F*) obtained from two-way ANOVA analysis in both sub-experiments are provided in Supplementary Table S2. Principal component analyses (PCAs) were performed using individual gene expression values and hormonal concentrations, and the default *prcomp* R function. Visualizations were constructed using the *factoextra* (v1.0.4) and *gplots* (v3.0.1) R package.

## Results

### Effects of Avt Administration

#### Plasma Osmolality

**FIGURE 1:**
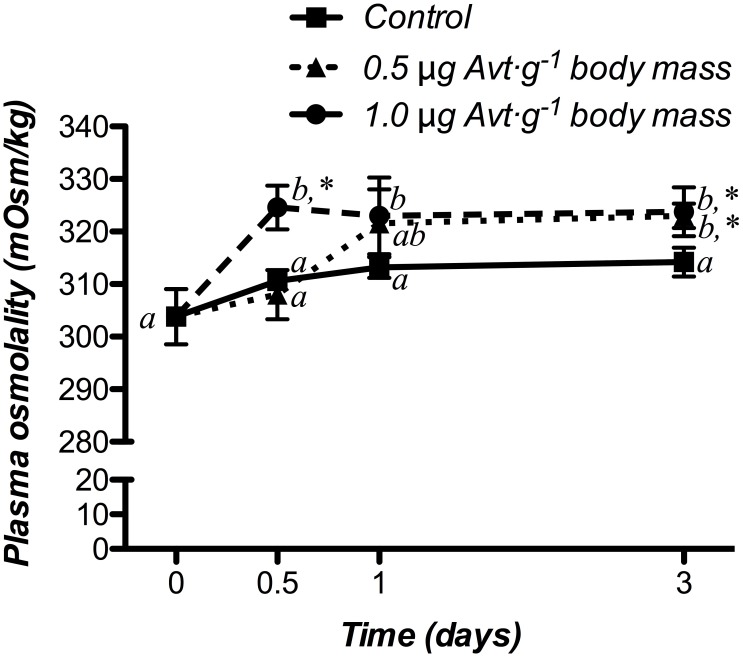
Time-course of changes in plasma osmolality in SW-adapted gilthead sea breams implanted with vegetable oil (mixture 5:1 coconut and sunflower oils) alone (control) or containing different doses of Arg^8^-vasotocin (Avt, 0.5 or 1 μg g^-1^ body mass). Values are represented as mean ± SEM (*n* = 7 fish per group). Significant differences among sampling points at the same Avt concentration are identified with different letters, whereas different symbols showed differences between groups at the same time (*P* < 0.05, two-way ANOVA followed by Tukey’s test).

Time-course changes in plasma osmolality response of sea bream juveniles to different doses of Avt are shown in Figure 1. In both doses, plasma osmolality increased its values 12 h (1.0 μg ⋅ g^-1^ body mass) or 3 days (0.5 μg ⋅ g^-1^ body mass) after treatment, maintaining statistically higher values compared to the control fish at the end of the experiment. In the control group, no significant changes were observed during the whole experiment.

#### Vasotocinergic and Isotocinergic Systems

**FIGURE 2:**
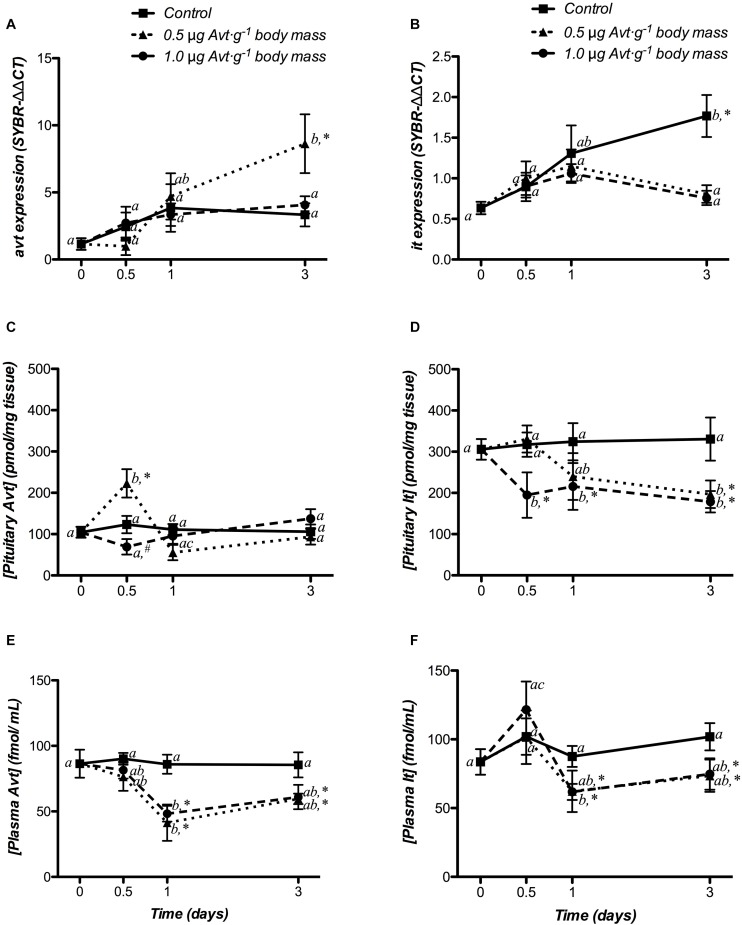
Time-course of changes in hypothalamic *avt* **(A)** and *it* **(B)** mRNA expression, hypophyseal Avt **(C)** and It **(D)** storage, and plasma Avt **(E)** and It **(F)** levels in SW-adapted gilthead sea breams implanted with vegetable oil (mixture 5:1 coconut and sunflower oils) alone (control) or containing different doses of Arg^8^-vasotocin (Avt, 0.5 or 1 μg g^-1^ body mass). Values are represented as mean ± SEM (*n* = 7 fish per group). Further details as described in the legend of Figure 1.

The time-course responses of hypothalamic *avt* and *it* expression to Avt treatment are shown in Figure 2A,B. In control fish, there were no variations in *avt* expression during the experiment, whereas *it* mRNA levels were significantly higher at day 3 post-treatment, when compared to non-treated fish (day 0). Avt treatment produced an increase in hypothalamic *avt* mRNA levels at the end of the experiment (day 3), but only at the lower dose of 0.5 μg ⋅ g^-1^ body mass. The mRNA expression of *it* did not show time-course variations after administration of both doses of Avt.

Changes in hypophyseal storage of Avt and It, in response to Avt treatment, are presented in Figure 2C,D. In the control group, Avt and It storage did not change during the experiment. Fish administered with 0.5 μg ⋅ g^-1^ of Avt increased the Avt storage in the pituitary 12 h post-treatment, when compared to non-treated fish (day 0), and then they recovered to control levels at day 1, with a strong interaction between both factors. It content showed a significant time- and dose-dependent decrease after 12 h (1.0 μg g^-1^ body mass of Avt) or 3 days (0.5 μg ⋅ g^-1^ body mass of Avt) of treatment, maintaining it until the end of the experiment with a significant interaction between both factors.

The time-course changes in plasma Avt and It levels after Avt treatment are shown in Figure 2E,F. In the control group, both nonapeptides did not significantly vary over time. However, in both Avt-treated groups, plasma Avt levels were significantly lower when compared to non-treated fish (day 0) at 24 h, or with respect to the control group from 24 h onward (Figure 2E). On the other hand, plasma It levels increased in animals treated with the highest dose of Avt (1.0 μg ⋅ g^-1^ body mass of Avt) 12 h post-administration, decreasing significantly from day 1 until the end of the experiment (Figure 2F). In addition, the administration of 0.5 μg g^-1^ body mass of Avt resulted in a significant time-dependent reduction of plasma It, with respect to the control group from day 1 onward (Figure 2F).

**FIGURE 3:**
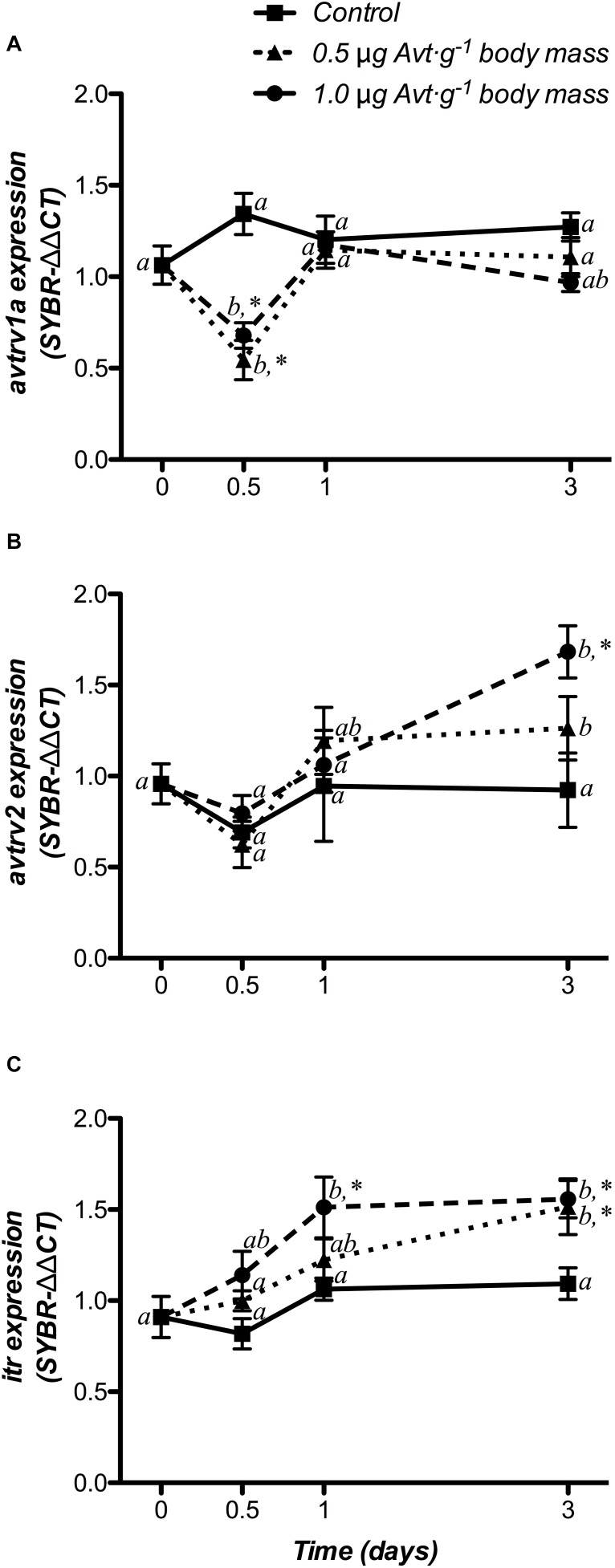
Time-course of changes on hypothalamic *avtrv1a* **(A)**, *avtrv2* **(B)**, and *itr* **(C)** mRNA expression in SW-adapted gilthead sea breams implanted with vegetable oil (mixture 5:1 coconut and sunflower oils) alone (control) or containing different doses of Arg^8^-vasotocin (Avt, 0.5 or 1 μg g^-1^ body mass). Values are represented as mean ± SEM (*n* = 7 fish per group). Further details as described in the legend of Figure 1.

Changes in Avt and It receptor gene expression (*avtrv1a*, *avtrv2*, and *itr*) in the hypothalamus are shown in Figure 3. There were no significant changes in *avtr*s or *itr* mRNA expression in the control group. However, *avtrv1a* significantly decreased during the first 12 h in both groups treated with 0.5 or 1.0 μg g^-1^ body mass of Avt, returning to baseline values after that (Figure 3A), showing a significant interaction between both factors. Moreover, at the end of the trial (day 3), *avtrv2* mRNA levels significantly increased in both Avt treated groups when compared to non-treated fish at day 0, with its highest expression after the administration of 1.0 μg g^-1^ body mass of Avt, with respect to the control fish (Figure 3B). In addition, a significant time- and dose-dependent increase was also observed for the *itr* mRNA levels from 1 day (1.0 μg g^-1^ body mass of Avt) or day 3 (0.5 μg g^-1^ body mass of Avt) after hormonal administration (Figure 3C).

#### HPI Axis

**FIGURE 4:**
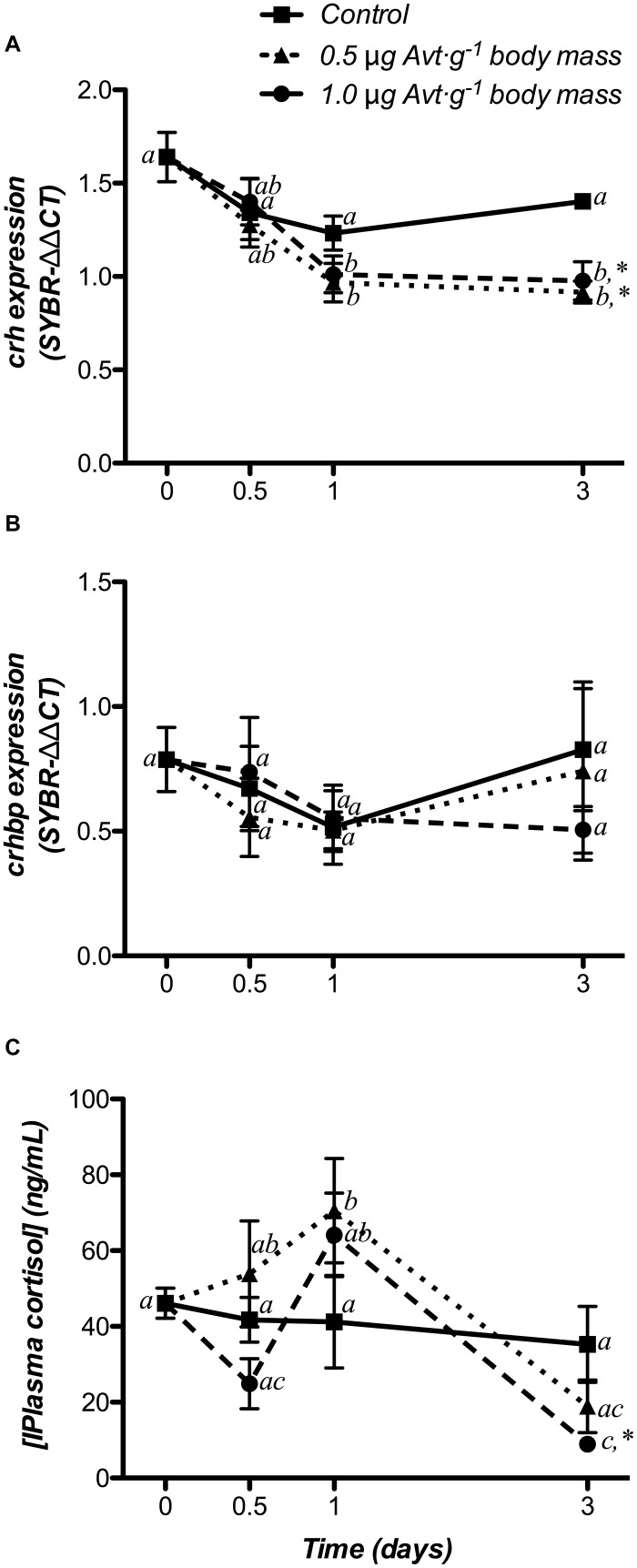
Time-course of changes on hypothalamic *crh* **(A)** and *crhbp* **(B)** mRNA expression, as well as on plasma cortisol levels **(C)**, in SW-adapted gilthead sea breams implanted with vegetable oil (mixture 5:1 coconut and sunflower oils) alone (control) or containing different doses of Arg^8^-vasotocin (Avt, 0.5 or 1 μg g^-1^ body mass). Values are represented as mean ± SEM (*n* = 7 fish per group). Further details as described in the legend of Figure 1.

Hypothalamic *crh* mRNA levels did not show variations over time in the control group, whereas Avt administration produced a significant decrease with respect to the non-treated fish (day 0) from day 1 onward in both 0.5 and 1.0 μg ⋅ g^-1^ body mass Avt treatments (Figure 4A), being also significantly lower when compared to the control group at the end of the trial. In addition, no variations in hypothalamic *crhbp* mRNA levels were observed in any of the three groups during the 3 days of the experiment (Figure 4B).

Plasma cortisol levels did not change in the control group but increased in fish administered with 0.5 μg ⋅ g^-1^ body mass at day 1 post-administration. In addition, treatment with 1.0 μg ⋅ g^-1^ body mass of Avt showed a significant decrease in plasma cortisol levels at day 3 (Figure 4C).

#### Thyroid System

**FIGURE 5:**
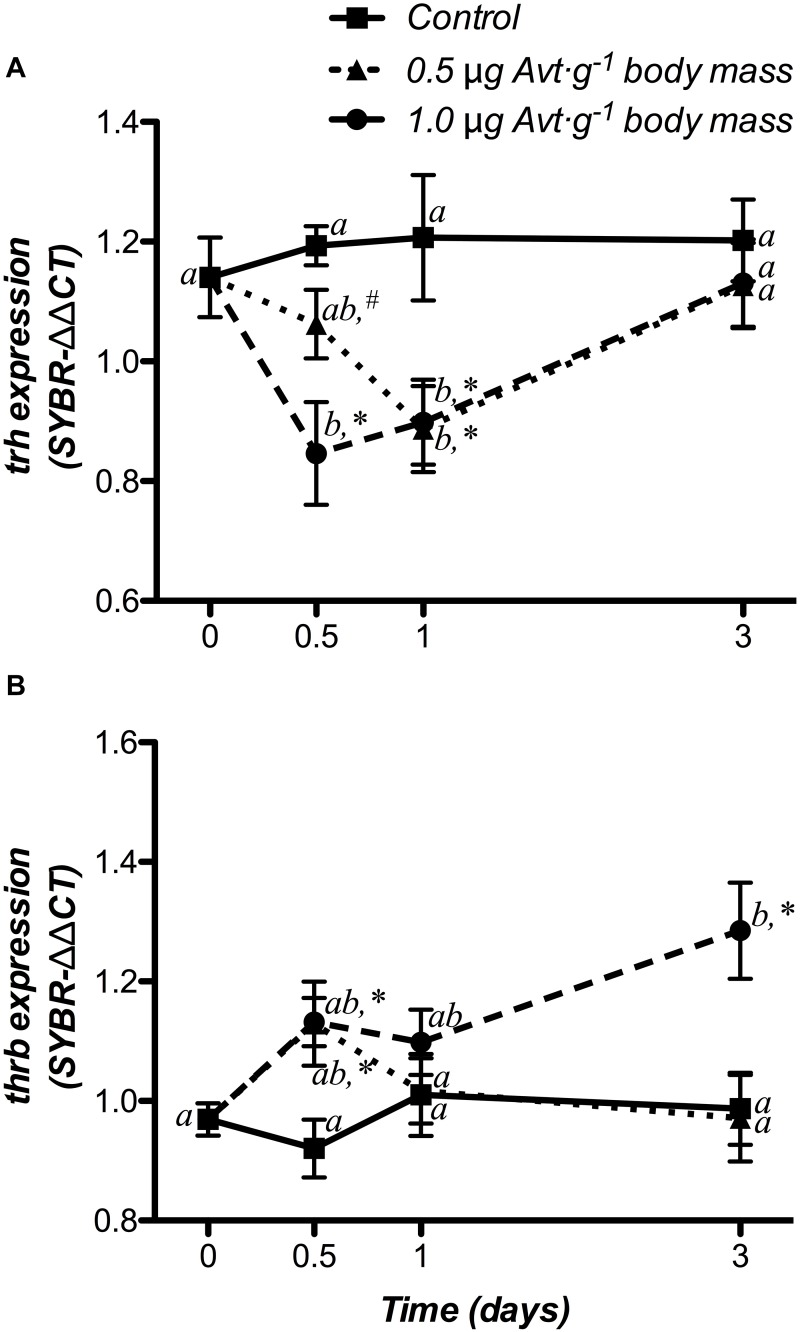
Time-course of changes on hypothalamic *trh* **(A)** and *thrb* **(B)** mRNA expression in SW-adapted gilthead sea breams implanted with vegetable oil (mixture 5:1 coconut and sunflower oils) alone (control) or containing different doses of Arg^8^-vasotocin (Avt, 0.5 or 1 μg g^-1^ body mass). Values are represented as mean ± SEM (*n* = 7 fish per group). Further details as described in the legend of Figure 1.

The time course responses of hypothalamic *trh* and *trβ* mRNA levels did not show variations in the control group (Figure 5). Both Avt doses (0.5 and 1 μg ⋅ g^-1^ body mass) produced a significant decrease in *trh* mRNA levels with respect to the non-treated fish (day 0) from 12 h to day 1 and also when compared to the control group, returning to control levels at day 3 post-administration (Figure 5A). A clear interaction between both factors showed a temporary increase in *trβ* mRNA expression only at 12 h post-administration in fish treated with 0.5 μg Avt ⋅ g^-1^ body mass, whereas those fish treated with 1.0 μg Avt ⋅ g^-1^ body mass maintained higher values during the 3 days that the experiment lasted (Figure 5B).

### Effects of Cortisol Administration

#### HPI Axis

**FIGURE 6:**
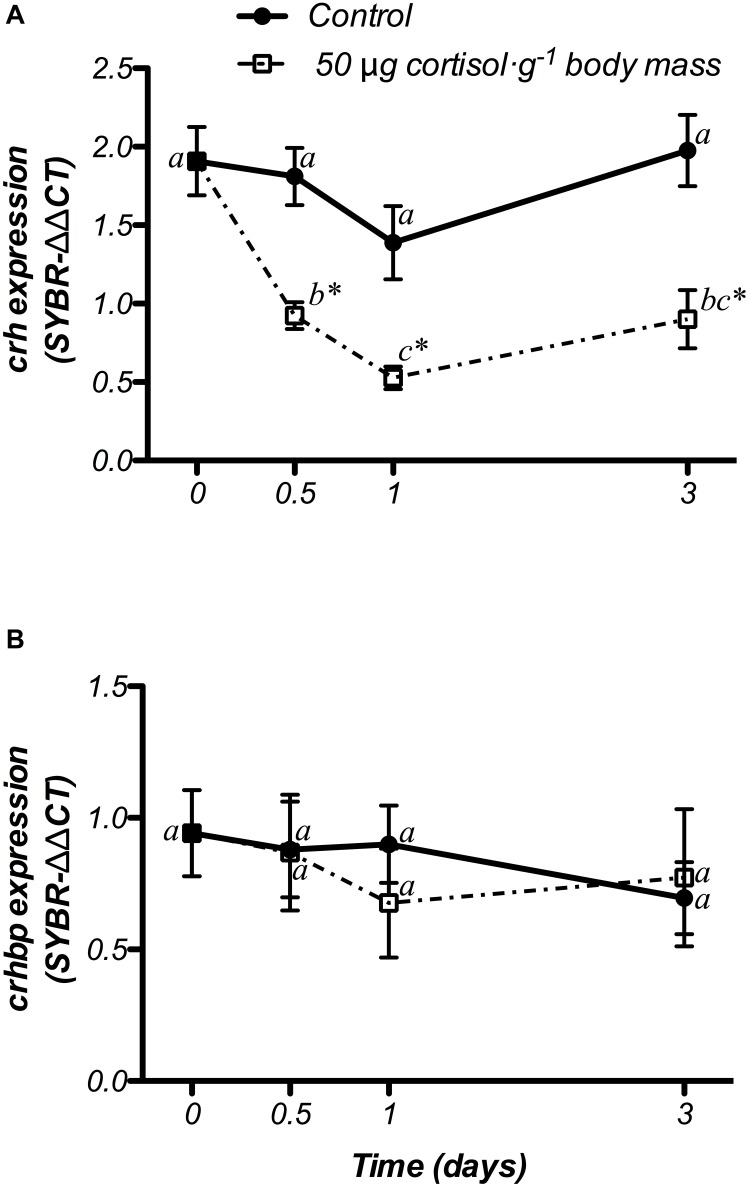
Time-course of changes on hypothalamic *crh* **(A)** and *crhbp* **(B)** mRNA expression in SW-adapted gilthead sea breams implanted with vegetable oil (mixture 5:1 coconut and sunflower oils) alone (control) or containing cortisol (50 μg g^-1^ body mass). Values are represented as mean ± SEM (*n* = 7–8 fish per group). Significant differences among sampling points at the same treatment (control or cortisol) are identified with different letters, whereas different symbols showed differences between groups at the same time (*P* < 0.05, two-way ANOVA followed by Tukey’s test).

Hypothalamic *crh* and *crhbp* mRNA changes after cortisol administration are presented in Figure 6. *crh* gene expression did not show significant variations in the control group, whereas 50 μg cortisol ⋅ g^-1^ body mass decreased its values from 12 h post-administration until the end of the experiment (3 days) when compared to both the non-treated (day 0) and the control fish (Figure 6A). Moreover, *crhbp* mRNA levels did not differ in the control and the cortisol-treated fish during the experiment (Figure 6B).

#### Thyroid System

**FIGURE 7:**
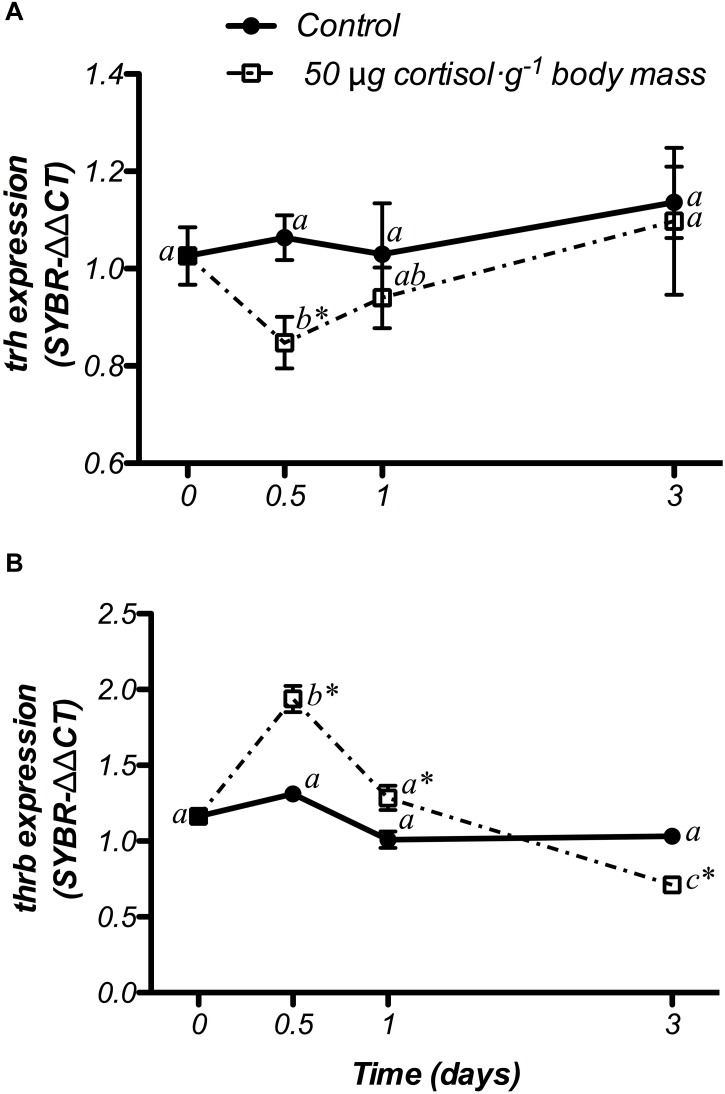
Time-course of changes on hypothalamic *trh* **(A)** and *thrb* **(B)** mRNA expression in SW-adapted gilthead sea breams implanted with vegetable oil (mixture 5:1 coconut and sunflower oils) alone (control) or containing cortisol (50 μg g^-1^ body mass). Values are represented as mean ± SEM (*n* = 7–8 fish per group). Further details as described in the legend of Figure 6.

Changes in *trh* and *thrb* mRNA expression at hypothalamic level are shown in Figure 7. The control fish did not show variations in either genes during the experiment. In addition, doses of 50 μg g^-1^ body mass of cortisol, down-regulated *trh* mRNA levels at 12 h post-treatment, when compared to both the non-treated (day 0) and control fish, returning to basal values after that (Figure 7A). Moreover, gene expression of *thrb* showed a significant increase at 12 h post-administration, which was statistically higher than both the non-treated fish (day 0) and the control group. Then, *thrb* mRNA levels decreased until the end of the trial, showing lower values at day 3 than those observed in control fish with a significant interaction between both factors (Figure 7B).

### PCA Analysis

**FIGURE 8:**
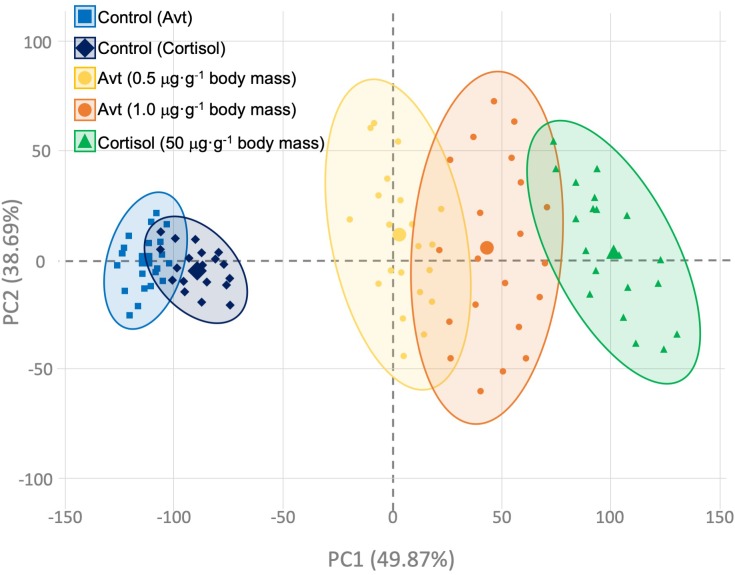
Principal component analysis (PCA) constructed using individual gene expression values and hormonal concentrations of those fish implanted with vegetable oil (mixture 5:1 coconut and sunflower oils) alone (control; blue squares and diamonds) or containing different doses of Arg^8^-vasotocin (0.5 μg Avt g^-1^ body mass, yellow circles; or 1 μg Avt g^-1^ body mass, orange circles) or cortisol (50 μg g^-1^ body mass, green triangles). Bigger symbols represent the centroids for each group’s ellipse.

The general PCA of all genes and hormonal parameters considered together, as well as the dataset previously published by our group (Cádiz et al., 2015) for the sub-trial related to cortisol injection, showed a clear separation in the two principal components, which explains in total 88.56% of the variability (Figure 8), where the effect of hormonal treatment (cortisol or Avt) seems to be explained by PC1 (49.87%), while the time-course effect appears to be more evident in PC2 (38.69%).

## Discussion

The putative regulation and interaction between the vasotocinergic, isotocinergic, HPI, and HPT axes has not been widely studied in fish (Larsen et al., 1998; Batten et al., 1999; Geven et al., 2006; Cádiz et al., 2015). Thus, the present study highlights the complex regulation of these endocrine pathways mediated by cortisol and Avt hormones. Slow-release implants of coconut oil containing Avt (0.5 or 1.0 μg ⋅ g^-1^ body mass) or cortisol (50 μg ⋅ g^-1^ body mass) successfully evoke changes in physiological parameters related to osmoregulation, stress, and metabolism in the gilthead sea bream (*S. aurata*) (Laiz-Carrión et al., 2002, 2003; Sangiao-Alvarellos et al., 2006; Cádiz et al., 2015). In fact, some of the up-stream players of the vasotocinergic and isotocinergic pathways, concomitantly with circulating levels of plasma cortisol, have recently been demonstrated to be affected by external cortisol administration in this model species (Cádiz et al., 2015). Moreover, as we will describe below for each sub-trial performed, it should be noted that our PCA analysis strongly suggests the cooperation of both Avt and cortisol endocrine players in terms of the direction of deviations with respect to the control groups, although the magnitude of changes seems to be produced in a more powerful way through cortisol administration (Figure 8). Thus, the clear parallelism described herein, regarding the stimulatory or inhibitory response independently caused by each hormone, reveals that their physiological actions can be considered to potentiate or trade-off the physiological action required in a complex net of molecular, biochemical, and endocrine interconnections, where the time-dependent effect caused by these hormones after treatment must be attended to in addition to the doses administered (see Supplementary Table S2).

It was not surprising to observe increased plasma osmolality in fish treated with Avt due to the well-known osmoregulatory function of hypothalamic nonapeptides such as Avt and AVP. Previous studies in gilthead sea bream treated with Avt and transferred to hypersaline water already showed an increase in their plasma osmolality levels (Sangiao-Alvarellos et al., 2006). Moreover, the higher osmolality observed herein in SW-acclimated fish in a time- and dose-dependent manner, demonstrate the activation of those mechanism needed during the adaptive period at the molecular and protein level for this hormone after salinity transfers (Martos-Sitcha et al., 2013b), were the contribution of ion absorption demonstrated *ex vivo* by Avt administration can be highly mediated by different portions of the intestine (i.e., anterior intestine and rectum) mainly achieved via the apical Na-K-2Cl (Nkcc2) co-transporter (Martos-Sitcha et al., 2013a). Our results also found an up-regulation of hypothalamic *avt* gene expression and hypophyseal Avt accumulation in fish administered with 0.5 μg g^-1^ body mass of Avt, which was not observed after the administration of 1 μg g^-1^ body mass of Avt. This finding suggests that administration of a low dose of Avt triggers the first step of the stress response, mediated by the vasotocinergic system during the adaptive period, which homeostasis can promptly restore through the combination of internal production and external administration of this hormone. The significant decrease in plasma Avt, in spite of its external administration, could be explained by a combination of (i) hormonal degradation by several peptidases, which present high activity levels in the plasma of the gilthead sea bream (Agirregoitia et al., 2005), (ii) its removal mediated by renal clearance due to its high concentration in the bloodstream (Amer and Brown, 1995), and (iii) its elimination by binding to specific receptors in target tissues to cope with physiological challenges. This last hypothesis is supported by the activation of the *avt receptors* (*avtrv1a* and *avtrv2*) in Avt treated fish. This happens not only at a central level (present results), but also in peripheral organs/tissues. In gills, opercular epithelium and the gastrointestinal tract, Avt receptors orchestrate several osmoregulatory functions related to ion transport and water absorption (Martos-Sitcha et al., 2013a, 2014a, 2015), whereas in the liver, gills, and the brain, metabolic changes related to energy repartitioning processes occurred during an activation of the vasotocinergic system (Sangiao-Alvarellos et al., 2004, 2006). This pattern of response in plasma Avt, as well as in the hypothalamic mRNA expression of both *avt receptors*, has also previously been observed in the gilthead sea bream after cortisol administration (Cádiz et al., 2015). Thus, the down-regulation observed in *avtrv1a* expression when these hormones (cortisol, Cádiz et al., 2015; or Avt, present results) are administrated, will mirror a stress situation induced by a hormonal challenge. This supports the role of the vasotocinergic system in the co-regulation of the HPI axis by negative feedback processes via *avtrv1a*, which can target the anterior pituitary, where Acth cells are also located (Antoni, 1984; Moons et al., 1989). Moreover, increased mRNA levels of *avtrv2* in the Avt-treated groups indicate that gene expression of this receptor is increased to co-regulate the HPI axis, as clearly shown by elevated plasma cortisol levels 24 h after Avt administration. In this regard, the participation of each pathway seems to be carried out sequentially, and our results reinforce the idea about the regulation of both (i) metabolic (Sangiao-Alvarellos et al., 2004, 2006) and (ii) cortisol cascade (Cádiz et al., 2015) pathways in the hypothalamus, highlighting an important upstream function at least during the first days of treatment. Moreover, the participation of both Avt receptors in the orchestration of hypothalamic function has recently been demonstrated after several stress-related challenges such as salinity transfer, high stocking densities, starving, or air exposure, where an activation of both vasotocinergic and HPI axes is produced (Martos-Sitcha et al., 2014a; Skrzynska et al., 2017, 2018a,b).

The present study also showed a decrease in It pituitary storage in the Avt-treated group 12–24 h post-administration. This finding, together with the absence of the up-regulation observed in *it* mRNA expression of control fish (Cádiz et al., 2015; present results), indicates that Avt administration activates the pituitary It secretion to modulate physiological functions in the periphery, also suggesting that a negative feedback can be produced in It neurons directly mediated by Avt in combination with It via Itr. Moreover, the subsequent decrease in plasma It concentration, together with the activation of *itr* mRNA expression at 12 h post-injection, suggests that plasma It previously secreted from the pituitary gland is being reduced by its binding to specific receptors. In previous studies performed in *S. aurata* juveniles intraperitoneally injected with cortisol, the decrease in plasma It levels was associated with an increase in the expression of the hypothalamic *itr* (Cádiz et al., 2015). The mechanism underlying this intriguing feature remains unclear, although a possible paracrine/autocrine pathway that interconnects both Avt/It systems, and/or a desensitization of Avt/It receptors to trade off the effect of each hormone when in excess, could not be ruled out. This last hypothesis has been observed in the opercular epithelium of fish, where the double physiological response observed to produce both absorptive and secretive functions in ion trafficking in a time-dependent manner mediated by different Avt receptors (Martos-Sitcha et al., 2015) is missed if It is applied 3-5 min before Avt (Martos-Sitcha and Fuentes, personal observation). Even so, the real binding capacity of Avt to Itr (or *vice versa*) will need further *in vivo* and *ex vivo* experimental approaches to be elucidated. However, taken together, our findings reinforce the idea of an interconnection between vasotocinergic and isotocinergic systems, as previously suggested in this (Cádiz et al., 2015) and other species (Batten et al., 1999), to modulate several physiological functions where these pleiotropic hormones are involved.

Previous works have provided indirect evidence for interconnections among Avt/It, HPI, and HPT axes. Axon profiles with immunoreactivity for the neurosecretory peptides vasotocin and isotocin formed large Herring bodies and terminal-like buttons in contact with corticotropic, growth hormone, thyrotropic, and *pars intermedia* cells (Batten et al., 1999). Our results clearly demonstrate that exogenous cortisol and Avt hormones produce the same type of response in both *crh* and *crhbp* gene expression, with a clear down-regulation of *crh* expression, and the absence of variations in *crhbp*. The similar responses confirm that both cortisol and Avt hormones strongly inhibit the HPI axis at the *crh* level by negative feedback processes, and hence the stress pathways are differentially sensitive to changes in several hormones expressed independently to perform the same physiological action. Moreover, the effect of cortisol injection in *crh* mRNA levels was stronger (fourfold decrease) than the response observed after Avt administration. This reinforces the idea that cortisol must be considered the key upstream player of the stress mediated response in fish (Wendelaar Bonga, 1997), in spite of the necessary support of the Avt system depending on the stress source (Mancera et al., 2008; Martos-Sitcha et al., 2013b; Cádiz et al., 2015). The absence of variations in *crhbp* expression also confirms that the HPI axis needs to be regulated by different pathways. Our results agreed with those of other authors who showed that *crhbp* expression remained unchanged in response to chronic stress when plasma cortisol levels were high (Wunderink et al., 2011). Moreover, the type (chronic or acute) and source (confinement, environmental, handling, etc.) of stress are very important factors to consider when this complex endocrine axis is examined (Martos-Sitcha et al., 2014b). Recently, it has been demonstrated that cortisol administration produced an important regulation in the vasotocinergic and isotocinergic systems in SW-acclimated gilthead sea breams (Cádiz et al., 2015). Thus, a potential interaction between the vasotocinergic, isotocinergic, and HPI axes was confirmed at the endocrine level through the strong correlation observed between plasma cortisol values and the synthesis (*avt* and *it* expression and pituitary storage of mature hormones) and release (plasma values) of Avt/It neuropeptides after hormonal (cortisol) treatment. Moreover, this experimental approach also displayed important effects in other peripheral tissues where Avt and/or It are also involved. Thus, the simulated stress situation produced by cortisol administration suggests a real cooperation to activate or trade off several physiological actions that can directly or indirectly be mediated by Avt and It receptors, such as those related to hepatic (and hypothalamic) metabolism, branchial regulation of ion trafficking by modulating the action of important ion pumps and transporters, such as Na^+^-K^+^-ATPase and Na-K-2Cl co-transporter, or even in feedback mechanisms that orchestrate the synthesis and release of cortisol in the interrenal tissue (see Laiz-Carrión et al., 2003; Sangiao-Alvarellos et al., 2006; Martos-Sitcha et al., 2014a, 2015; Cádiz et al., 2015 for review).

In the same way, Avt treatment also produced a transitory increase of plasma cortisol, revealing a first release suppression with the highest dose, followed by a withdrawal and reduction of its levels at the end of the regulatory period (3 days post-treatment). Cortisol was shown to effect self-suppression by negative feedback of its secretion directly at the level of the interrenal gland (Bradford et al., 1992), whereas its regulation could also be carried out at the peripheral level, at least in part, by Avt and/or It receptor genes in this tissue (Cádiz et al., 2015), as strongly suggested by the present results.

Our results also indicate that Avt and cortisol regulates the HPT axis. Both hormonal treatments affected hypothalamic *trh* and *thrb*, up-regulating and down-regulating their mRNA expression, respectively. In vertebrates, including fish, Trh acts as a multifunctional hypophysiotropic regulator, stimulating important factors such as Acth and α-Msh (Wendelaar Bonga, 1997; Galas et al., 2009). Cortisol and Avt, together with thyroidal hormones, have been demonstrated to participate and share several physiological functions, such as those adaptations caused by several situations, such as osmoregulatory challenges. Altogether, a complex network of interconnections cannot be ruled out, as it has previously been shown for cortisol (Wendelaar Bonga, 1997; Laiz-Carrión et al., 2003; Cádiz et al., 2015) and Avt (Janssens and Lowrey, 1987; Moon and Mommsen, 1990; Sangiao-Alvarellos et al., 2006). Thus, the orchestration of overall physiology by these endocrine players has been historically demonstrated to cause metabolic arrangements related to hyperglycemia, lipid, and protein mobilization (reviewed by Plisetskaya et al., 1983) aimed to activate catabolic routes by operating energy reserves as a secondary stress response in teleost fish (Barton and Iwama, 1991). In addition, these hormonal pathways also affect ion regulation mediated by osmoregulatory peripheral tissues (Sangiao-Alvarellos et al., 2006; Arjona et al., 2008, 2011; McCormick et al., 2008; Ruiz-Jarabo et al., 2017, 2018). Our results support a feedback mechanism in which cortisol and Avt act to reduce the activity of hypothalamic Trh, thus potentially integrating thyrotropic function, which can be achieved up-stream by the *thrb* in addition to the other factors and receptors described above.

## Conclusion

**FIGURE 9:**
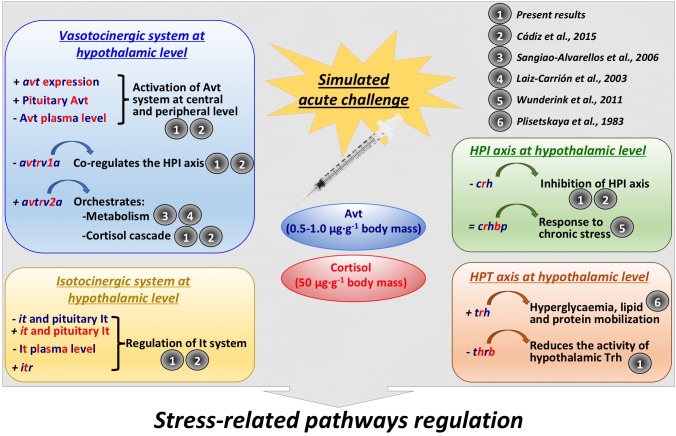
Summary of the interactions found in the present study, as well as further references that support them, between vasotocinergic, isotocinergic, HPI, and HPT pathways at hypothalamic level. Dark blue: Avt effect; red: cortisol effect; dark blue and red: effect produced by both hormones.

As summarized in Figure 9, this study identified for the first time new evidence regarding an interaction among the vasotocinergic, isotocinergic, interrenal, and thyroid pathways at the hypothalamic level, whose up-stream regulation seems to be orchestrated, at least in part, by the final products of some of them, such as cortisol and Avt. In addition, the interconnection of all of them seems to be thanks to a close participation of specific receptors only present and active at the hypothalamic level which is strongly suggested by the PCA performed. This will modulate the final hormonal release and functioning to trigger and achieve the physiological action required in peripheral tissues, such as metabolism, osmoregulation, and stress-related processes. It is important to closely control this intriguing relationship through a complex net of different hormones and factors synthetized independently, which will trade off and avoid an overload of the physiological function required, since all of them are involved through direct or indirect mechanisms. However, the precise mechanism involved in this complex network of relationships and the way in which these communications are performed remains unclear, and future analyses are needed to determine whether the final biological function is produced after a specific stimulation or inhibition of these endocrine systems.

## Author Contributions

JM-S and JM conceived and designed the study. JM-S, LC, and GM-R carried out experimental procedures and analyzed transcriptomic data. MG and EK analyzed Avt and It content in plasma and pituitary. JM-S, LC, and JM wrote the original draft. All authors interpreted the data and reviewed, edited, and approved the final manuscript.

## Conflict of Interest Statement

The authors declare that the research was conducted in the absence of any commercial or financial relationships that could be construed as a potential conflict of interest.

## Footnotes

1 https://wiki.zfin.org/display/general/ZFIN+Zebrafish+Nomenclature+Guidelines
