# Supplementary material for: Arginine Vasotocin and Cortisol Co-regulate Vasotocinergic, Isotocinergic, Stress, and Thyroid Pathways in the Gilthead Sea Bream (Sparus aurata)
Source: Front Physiol. 2019 Mar 21;10:261. doi: 10.3389/fphys.2019.00261 (PMC6437069; doi:10.3389/fphys.2019.00261)
Supplement: Supplementary file 1 [file Table_1.DOCX]

**Table 1.** Specific primers used for semi-quantitative expression by PCR (qPCR), primer concentration used, and the amplified size for each pair of primers.

| ***Primers*** | ***Nucleotide sequence*** | ***Primer concentration*** | ***Amplicon size*** |
| --- | --- | --- | --- |
| **qPCR-*avt*_F_** | 5’-agaggctgggatcagacagtgc-3’ | 200 nm | 129 bp |
| **qPCR-*avt*_R_** | 5’-tccacacagtgagctgtttccg-3’ |  |  |
| **qPCR-*it*_F_** | 5’-ggagatgaccaaagcagcca-3’ | 200 nm | 151 bp |
| **qPCR-*it*_R_** | 5’-caaccatgtgaactacgact-3’ |  |  |
| **qPCR-*avtrv1a*_F_** | 5’-gacagccgcaagtgatcaag-3’ | 400 nm | 203 bp |
| **qPCR-*avtrv1a*_R_** | 5’-cccgaccgcacaccccctggct-3’ |  |  |
| **qPCR-*avtrv2*_F_** | 5’-atcacagtccttgcattggtg-3’ | 600 nm | 120 bp |
| **qPCR-*avtrv2*_R_** | 5’-gcacaggttgaccatgaacac-3’ |  |  |
| **qPCR-*itr*_F_** | 5’-ggaggatcgttttaaagacatgg-3’ | 400 nm | 120 bp |
| **qPCR-*itr*_R_** | 5’-tgttgtctccctgtcagattttc-3’ |  |  |
| **qPCR-*crh*_F_** | 5’-atggagaggggaaggaggt-3’ | 200 nm | 176 bp |
| **qPCR-*crh*_R_** | 5’-atctttggcggactggaaa-3’ |  |  |
| **qPCR-*crhbp*_F_** | 5’-gcagcttctccatcatctacc-3’ | 200 nm | 147 bp |
| **qPCR-*crhbp*_R_** | 5’-acgtgtcgataccgcttcc-3’ |  |  |
| **qPCR-*trh*_F_** | 5’-gaaacgcttttgggataactcc-3’ | 300 nm | 131 bp |
| **qPCR-*trh*_R_** | 5’-cggcgtgactcttgtttatgtt-3’ |  |  |
| **qPCR-*thrb*_F_** | 5’-atactgtgacccgaccctctaa-3’ | 200 nm | 120 bp |
| **qPCR-*thrb*_R_** | 5’-attatctctggaacgcagcact-3’ |  |  |
| **qPCR-*actb*_F_** | 5’-tcttccagccatccttcctcg-3’ | 200 nm | 108 bp |
| **qPCR-*actb*_R_** | 5’-tgttggcatacaggtccttacgg-3’ |  |  |
