# Supplementary material for: Arginine Vasotocin and Cortisol Co-regulate Vasotocinergic, Isotocinergic, Stress, and Thyroid Pathways in the Gilthead Sea Bream (Sparus aurata)
Source: Front Physiol. 2019 Mar 21;10:261. doi: 10.3389/fphys.2019.00261 (PMC6437069; doi:10.3389/fphys.2019.00261)
Supplement: Supplementary file 2 [file Table_2.DOCX]

**Suppl. Table 2.** Statistical parameters (P-value and F) obtained from two-way ANOVA analysis in fish treated with different doses of hormones in a short-time response.

| **Parameter** | **[Avt]** | | **Time** | | **Interaction** | |
| --- | --- | --- | --- | --- | --- | --- |
|  | **P-value** | **F** | **P-value** | **F** | **P-value** | **F** |
| Osmolality | **0.0325** | 4.125 | **0.0001** | 8.204 | 0.4427 | 0.9888 |
| *avt* | 0.0932 | 1.934 | **<0.0001** | 9.211 | 0.0963 | 1.922 |
| *It* | **0.0377** | 3.523 | **0.0012** | 6.238 | 0.0701 | 2.111 |
| Pituitary Avt | 0.4606 | 0.789 | **0.0234** | 3.482 | **<0.0001** | 6.414 |
| Pituitary It | **0.0050** | 5.997 | **0.0315** | 2.976 | **0.0455** | 2.616 |
| Plasma Avt | **0.0074** | 5,462 | **0.0028** | 5.441 | 0.3297 | 1.188 |
| Plasma It | 0.0705 | 2.844 | **0.0049** | 4.864 | 0.4672 | 0.9524 |
| *avtrv1a* | **0.0006** | 8.696 | **0.0019** | 5.733 | **0.0018** | 4.164 |
| *avtrv2* | **0.0438** | 4.371 | **0.0007** | 6.701 | 0.2645 | 1.323 |
| *itr* | **0.0019** | 7.123 | **<0.0001** | 11.73 | 0.2886 | 1.270 |
| *crh* | **0.0444** | 3.316 | **<0.0001** | 16.52 | 0.3121 | 1.219 |
| *crhbp* | 0.8833 | 0.1244 | 0.3181 | 1.209 | 0.9192 | 0.3269 |
| Cortisol | 0.0911 | 2.472 | **0.0003** | 7.830 | 0.1222 | 1.801 |
| *trh* | **0.0025** | 6.818 | **0.0237** | 3.448 | 0.0873 | 1.979 |
| *thrb* | **0.0009** | 8.162 | **0.0418** | 2.760 | **0.0171** | 2.885 |
|  | | | | | | |
| **Parameter** | **[Cortisol]** | | **Time** | | **Interaction** | |
|  | **P-value** | **F** | **P-value** | **F** | **P-value** | **F** |
| *crh* | **<0.0001** | 28.38 | **0.0001** | 8.439 | **0.0275** | 3.276 |
| *crhbp* | 0.7704 | 0.086 | 0.7176 | 0.4512 | 0.8815 | 0.2207 |
| *trh* | **0.0466** | 3.169 | **0.0389** | 3.913 | 0.1440 | 1.969 |
| *thrb* | **0.0007** | 12.82 | **<0.0001** | 62.53 | **<0.0001** | 25.46 |
